# Comparative transcriptomic analysis reveals the underlying molecular mechanism in high-fat diet-induced islet dysfunction

**DOI:** 10.1042/BSR20230501

**Published:** 2023-07-04

**Authors:** Shengrong Wan, Ying An, Wei Fan, Fangyuan Teng, Zongzhe Jiang

**Affiliations:** 1 Experimental Medicine Center, The Affiliated Hospital of Southwest Medical University, Luzhou, Sichuan 646000, China; 2 Metabolic Vascular Disease Key Laboratory of Sichuan Province, Sichuan 646000, China; 3 Academician (Expert) Workstation of Sichuan Province, The Affiliated Hospital of Southwest Medical University, Luzhou, Sichuan 646000, China; 4 Department of Endocrinology and Metabolism, The Affiliated Hospital of Southwest Medical University, Luzhou, Sichuan 646000, China; 5 Department of Orthopaedics, The Affiliated Hospital of Southwest Medical University, Luzhou, Sichuan 646000, China

**Keywords:** High fat diet, islet dysfunction, Obesity, RNA-seq

## Abstract

Obesity, characterized by accumulation of adipose, is usually accompanied by hyperlipidemia and abnormal glucose metabolism, which destroys the function and structure of islet β cells. However, the exact mechanism of islet deterioration caused by obesity has not yet been fully elucidated. Here, we fed C57BL/6 mice with a high-fat diet (HFD) for 2 (2M group) and 6 months (6M group) to construct obesity mouse models. Then, RNA-based sequencing was used to identify the molecular mechanisms in HFD-induced islet dysfunction. Compared with the control diet, a total of 262 and 428 differentially expressed genes (DEGs) were identified from islets of the 2M and 6M groups, respectively. GO and KEGG enrichment analysis revealed that the DEGs up-regulated in both the 2M and 6M groups are mainly enriched in response to endoplasmic reticulum stress and the pancreatic secretion pathway. DEGs down-regulated in both the 2M and 6M groups are mainly enriched in the neuronal cell body and protein digestion and absorption pathway. Notably, along with the HFD feeding, mRNA expression of islet cell markers was significantly down-regulated, such as *Ins1, Pdx1, MafA* (β cell), *Gcg, Arx* (α cell), *Sst* (δcell), and *Ppy* (PP cell). In contrast, mRNA expression of acinar cell markers was remarkably up-regulated, such as *Amy1, Prss2*, and *Pnlip*. Besides, a large number of collagen genes were down-regulated, such as *Col1a1, Col6a6*, and *Col9a2*. Overall, our study provides a full-scale DEG map regarding HFD-induced islet dysfunction, which was helpful to understand the underlying molecular mechanism of islet deterioration further.

## Introduction

As a chronic metabolic disease caused by diverse factors, obesity is usually accompanied by hyperlipidemia and abnormal glucose metabolism [1,2], which could damage the function and morphology of pancreatic islet β cells and affect the synthesis and secretion of insulin, leading to the development of T2DM [3–5]. With the progress of society and the change in people’s diet and lifestyle, the number of obese patients is increasing with each passing day, and the probability of metabolic syndrome (MetS) in obese patients is significantly increased [6]. Although the typical characteristics of MetS, such as obesity, hypertension, and hyperlipidemia, are recognized as risk factors for diabetes mellitus, the molecular mechanism of the structural and functional damage of pancreatic islets caused by obesity has not been fully elucidated.

The pathogenesis of obesity includes genetic and environmental factors, including a high-fat diet, which can cause a variety of chronic diseases including diabetes mellitus, cardiovascular diseases, obstructive sleep apnea syndrome, impaired fertility function, and various cancers [7]. Insulin resistance and pancreas islet dysfunction, especially β-cell reduction, are reported to contribute to obesity-induced T2DM [8]. Some studies have shown that due to the imbalance of energy metabolism, obese individuals will present a low degree of inflammation in adipose, muscle, and liver tissue, especially in pancreatic islet, which leads to impaired islet function and promote the occurrence and development of T2DM [9,10]. However, hyperinsulinemia under T2DM will in turn lead to islet β-cell dysfunction, eventually leading to massive loss of β cells through apoptosis, aggravating the damage of pancreatic islet function [11,12]. Previous research explored the changes of mRNA and protein in islets of Goto-Kakizaki (GK) rats through transcriptome sequencing and proteomics. They found that the early stage (4–6 weeks) of islets of GK rats was characterized by anaerobic glycolysis, inflammation initiation, and compensation of insulin synthesis, and the late stage (8–24 weeks) was characterized by inflammation amplification and compensation failure. [13]. It can be seen that the use of transcriptome-oriented research is critical to investigate the gene changes that occur in pancreatic islets that are mainly composed of β cells. β-cell dysfunction during MetS progression is a gradual process [14], however, many studies have focused on analyzing gene changes of the disease at a single time point. Therefore, it is meaningful to conduct studies to explore the evolution of molecular events in pancreatic islets at various time points of obesity.

In this study, to search the characteristics of pancreatic islets injury caused by obesity at the molecular level, we used the RNA sequencing (RNA-seq) technique to generate integrated transcriptome profiles of pancreatic islets in C57BL/6 mice after the establishment of obesity (HFD-induced in 2 and 6 months). Our study provides insight into the molecular mechanism by which obesity leads to structural and functional impairment of pancreatic islets and identifies potential treatment targets for obesity-induced islet cell dysfunction.

## Materials and methods

### Animals

All animal experiments were performed in the Animal Experiment Center of Southwest Medical University. Five-week-old male C57BL/6 mice (*n*=15) were purchased from the Nanjing Biomedical Research Institute of Nanjing University (Nanjing, China). Animals were acclimatized before the experiments for at least one week, then they were randomly divided into three groups (Control; HFD-2M; HFD-6M; *n*=5 in each group). Normal chow diet (1022) containing 20% of calories from fat, 23% from protein, and 57% from carbohydrates was purchase from Beijing Huafukang Biological Technology Co., Ltd. (Beijing, China). HFD containing 60% of calories from fat, 20% from protein, and 20% from carbohydrates was purchased from Research Diets (D12492, USA). Blood glucose was measured by Accu-check glucometer. After 6 months, the mice were anesthetized by intraperitoneal injection of 0.5% pentobarbital sodium for sample collection. In addition, mice were sacrificed through a carbon dioxide release device. Animal experiments were approved by the Institutional Animal Ethics Committee of Southwest Medical University (Luzhou, China, No.20210819-91) and in accordance with the National Institutes of Health (NIH) guidelines for the care and use of laboratory animals.

### Oral glucose tolerance test

For the oral glucose tolerance test (OGTT), the mice have gavaged 20% glucose (1 g/kg) after collection of blood at baseline (0 min). All blood samples were collected from the tail vein at 0, 15, 30, 60, and 120 min for 20% glucose measurements.

### Islets isolation and purification

1. Prepare 2 mg/ml collagenase P (*Roche Life Science, Indianapolis, IN*) with ice cold 1X HBSS; using 6 ml per mouse. And prepare 1X HBSS containing 10% FBS as the STOP solution, keep on ice.
2. The donor mice were anesthetized by intraperitoneal injection of 0.5% pentobarbital sodium, and after confirming the mouse is deeply anesthetized, euthanasia was performed by dislocating cervical vertebrae.
3. Secure the mouse on an absorbent pad in a supine position. Disinfect the abdominal skin using 75% ethanol. Open the abdominal cavity and gently move the intestines to the right to expose the bile duct, hepatic artery, and pancreas.
4. Identify the Vater ampulla, insert a No. 4.5 needle connected to a syringe containing 3 ml of collagenase P solution into the Vater ampulla and slowly inject collagenase P into the common bile duct, forcing it to enter the pancreatic duct to fully inflate the pancreas.
5. Carefully remove the inflated pancreas and place it in a 50 ml centrifuge tube containing 3 ml of pre-cooled collagenase P solution. Fix the tube in a 37°C water bath for digestion for approximately 14–15 min while shaking every 2 min. After incubation, manually shake the tube until the pancreas has been transformed into fine sand-like particles, then immediately place the tube on ice and add 6 times the volume of cold STOP solution to terminate digestion.
6. Centrifuge the tube at 4°C, 1200 rpm for 2 min to discard the supernatant, and add 20 ml of STOP solution into each tube to mix evenly, centrifuge again under the same conditions, discard the supernatant and wash twice.
7. Resuspend precipitation with pre-cooled 1X HBSS at 4°C, 1200 rpm, centrifuge for 2 min, discard the supernatant and wash twice.
8. Add 8 ml of density gradient to the 50 ml tube and vortex briefly at low speed with the precipitate until homogenized. Then, gently add another 8 ml of density gradient along the wall to the tube. Finally, drip 8 ml of room temperature 1× HBSS on the upper layer, centrifuge tube at 10°C, 2400 rpm for 30 min. Make sure that the acceleration/deceleration speed is changed to the lowest setting to maintain the gradient.
9. Transfer the suspended particles in the middle of the tube to a new 50 ml centrifuge tube. Add 20 ml of pre-cooled 1× HBSS and centrifuge at 4°C, 1000 rpm for 2 min. Discard the supernatant and wash twice.
10. Add 6 ml preheat RPMI-1640 medium (10% FBS, 1% penicillin/ streptomycin) to the precipitated particles, and transferred this mixture to a culture dish. Then, pancreatic islets were selected under a microscope. Finally, incubate the islets in a 37°C, 5% CO_2_ incubator for cultivation or extract total mRNA for experimental purposes.

### cDNA library construction and sequencing

The total RNA of pancreatic islets (*n* = 3 for the Control, HFD-2M, and HFD-6M groups, respectively) was extracted separately using Trizol Reagent (Invitrogen, CA, U.S.A.), following the manufacturer's procedure. The integrity of the total RNA sample was tested by agarose gel electrophoresis, and *Nanodrop ND-1000* (Thermo Fisher Scientific, Waltham, MA, U.S.A.) was used for quantification and further quality inspection. Total RNA for 1–2 µg was selected from each sample and *NEB Next® Poly (A) mRNA Magnetic Isolation Module* (New England Biolabs) was used to enrich mRNA. The enriched mRNA was used to construct the library with *KAPA Stranded RNA-Seq Library Prep Kit* (Illumina). The constructed library was inspected by *Agilent 2100 Bioanalyzer* (Agilent Technologies, Inc., CA, U.S.A.), and the final quantification of the library was performed by qPCR.

The sequencing library of mixed different samples was denatured with 0.1 M NaOH to generate single-stranded DNA, which was diluted to 8 pM for situ amplification by *TruSeq SR Cluster Kit v3-cBot-HS* (#GD-401-3001, Illumina). The end of the amplified DNA fragments was sequenced on *Illumina HiSeq 4000* (LC Sciences, U.S.A.) at *Aksomics Inc*. (Shanghai, China), and the sequencing reaction was run for 150 cycles.

### Principal component and correlation analysis of gene expression level

We performed principal component (PC) analysis on genes with a significant mean difference (ANOVA, *P* ≤ 0.05) in all samples to obtain the intuitive distribution of samples between the experimental group and the control group. Pearson correlation coefficients were used to analyze the correlation of gene expression between samples. The closer the value is to one, the higher the similarity between the two samples.

### Differential expression genes analysis

Analyzing the expression of differential expression genes (DEGs) requires quantification of the expression level. The gene expression was normalized by the method of Fragments Per Kilobases per Millionreads [15]. The resulting *P* values were accustomed to following Benjamini and Hochberg’s method [16] for regulating the false discovery rate (FDR). Genes were represented as DEGs when the adjusted *P* values were < 0.05 and |log2 (fold change) | ≥ 1.5. Then we conducted a hierarchical cluster analysis of DEGs and drew a gene expression volcano plot.

### Functional enrichment analysis of mRNA

GO was used to classify gene functions and describe the molecular function, cellular component, and biological processes of each gene. Goatools software (Version 0.6.5) [17] was used for GO enrichment analysis. To further analyze the pathways involved by DEGs, the KEGG database (Version 2017.08) [18,19] was used to enrich and analyze the pathways with significant changes. Categories with FDR < 0.05 were considered significant GO terms and KEGG pathways.

### Immunofluorescence staining

For immunofluorescence staining, slides of mouse pancreas were stained with anti-insulin antibody (1:200 dilution, cst#4590, Cell Signaling Technology, U.S.A.), anti-glucagon antibody (1:200 dilution, cst#2760, Cell Signaling Technology, U.S.A.) and anti-*Col1a1* antibody (1:200 dilution, sc293182, Santa Cruz, U.S.A). FITC and Cy3 fluorescent dye-conjugated secondary antibodies (1:200 dilution, Biosynthesis Biotech, China) were incubated for 60 min at room temperature in the dark. The nucleus was labeled with DAPI, and images were taken with a fluorescence microscope (Leica, Germany).

### Statistical analysis

In the animal experiments, data are expressed as the means ± standard deviation (SD). The Student’s *t*-test was employed for comparison between the two groups. Differences were evaluated using GraphPad Prism 8. *P*<0.05 was considered statistically significant. *, *P*<0.05; **, 0.001 < *P* < 0.01; ***, *P*<0.001; #, *P*<0.05, ###, *P*<0.001.

## Results

### High-fat diet-induced islet dysfunction in obesity mouse model

**Figure 1:**
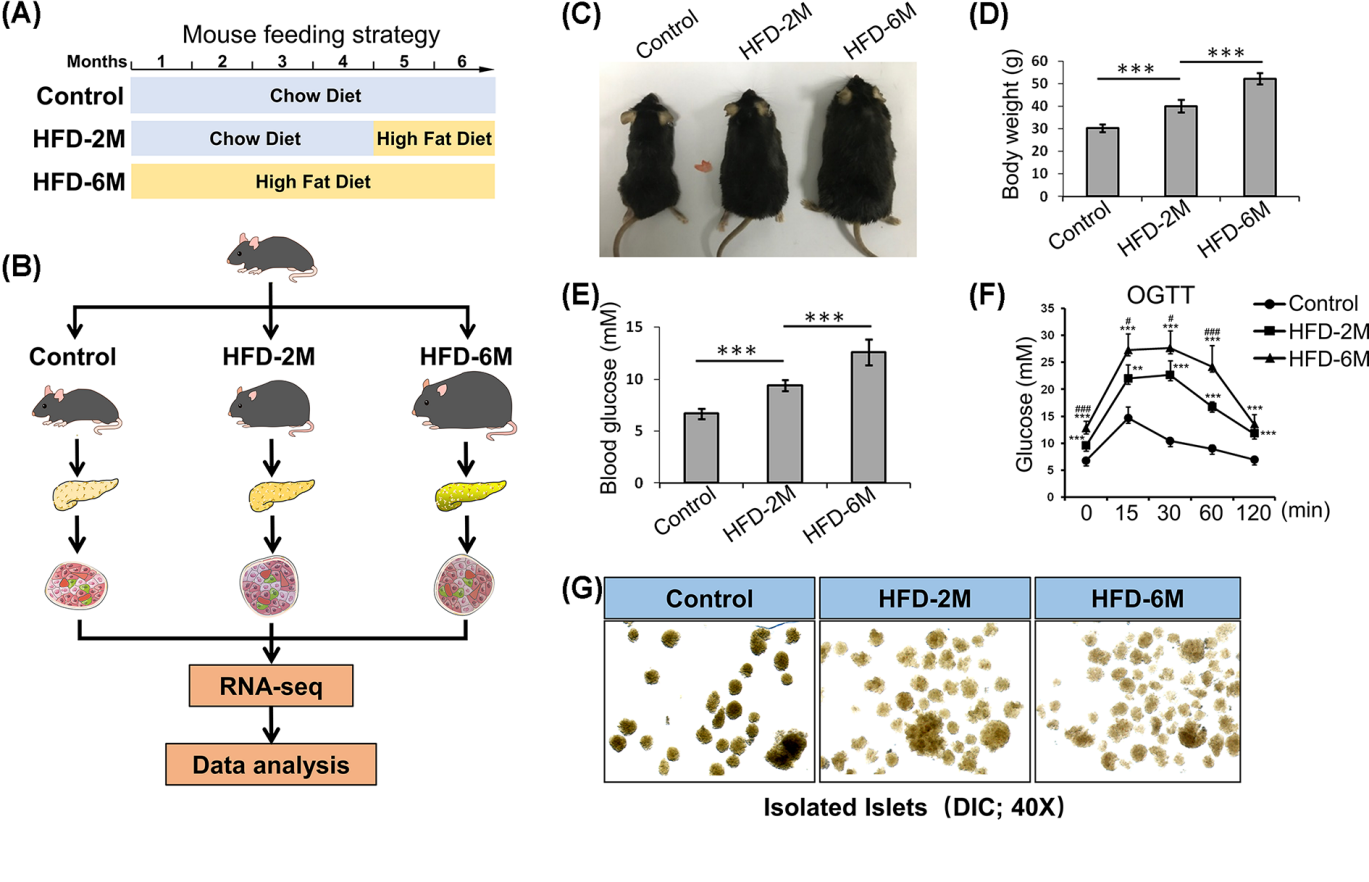
HFD-induced obesity and islet dysfunction in C57BL/6 mice (**A**) The mouse feeding strategy. (**B**) Experimental workflow. Pancreatic islets isolated from Control, HFD-2M, and HFD-6M group were subjected to RNA-seq. After performing data quality control and normalization, differentially expressed mRNAs were analyzed by ANOVA followed by integrated bioinformatics analysis and biological validation. (**C**) Representative images of mice in the Control, HFD-2M, and HFD-6M groups. (**D**) Body weight of mice fed with HFD for 2 and 6 months. Littermates fed with a chow diet were used as controls. Data represent mean ± SD; ***, *P*<0.001; two-tailed unpaired Student’s *t* test. (**E**) Fasting blood glucose levels in mice with indicated treatment. Data represent mean ± SD; ***, *P*<0.001; two-tailed unpaired Student’s *t* test. (**F**) OGTT of Control, HFD-2M, and HFD-6M group (*n* = 5 in each group). Data represent mean ± SD. **P<0.01, ***P<0.001 compared with control group at the same time point; #*P*<0.05, ###*P*<0.001 compared with HFD-2M group at the same time point; two-tailed unpaired Student’s *t* test. (**G**) 40X DIC representative islets images of Control, HFD-2M, and HFD-6M mice. DIC, differential interference contrast; HFD, high-fat diet; OGTT, oral glucose tolerance test; SD, standard deviation.

As shown in Figure 1A, to investigate the transcriptome features of pancreatic islets in obesity, we first established obesity mouse models through HFD feeding for 2 months (HFD-2M) or 6 months (HFD-6M). Then the islets were isolated for RNA-seq assay (Figure 1B). Compared with the control mice, the body weight of C57BL/6 mice with HFD feeding increased significantly (Figure 1C,D). Similarly, fasting blood glucose levels were also elevated along with the HFD feeding (Figure 1E). Besides, the oral glucose tolerance test (OGTT) showed that the glucose tolerance of HFD-fed mice was remarkably impaired, especially in HFD-6M mice (Figure 1F). These data suggested that the obesity mouse model was constructed successfully and the function of the islet from HFD-fed mice was impaired. Then we isolated islets from the pancreases of HFD-fed mice and indicated control mice. Of note, with differential interference contrast (DIC) microscopy, we found that the pancreatic islets of HFD-fed mice showed a disturbed morphology with irregular shape and lightened color compared with control mice (Figure 1G).

### mRNA sequencing and analysis

**Figure 2:**
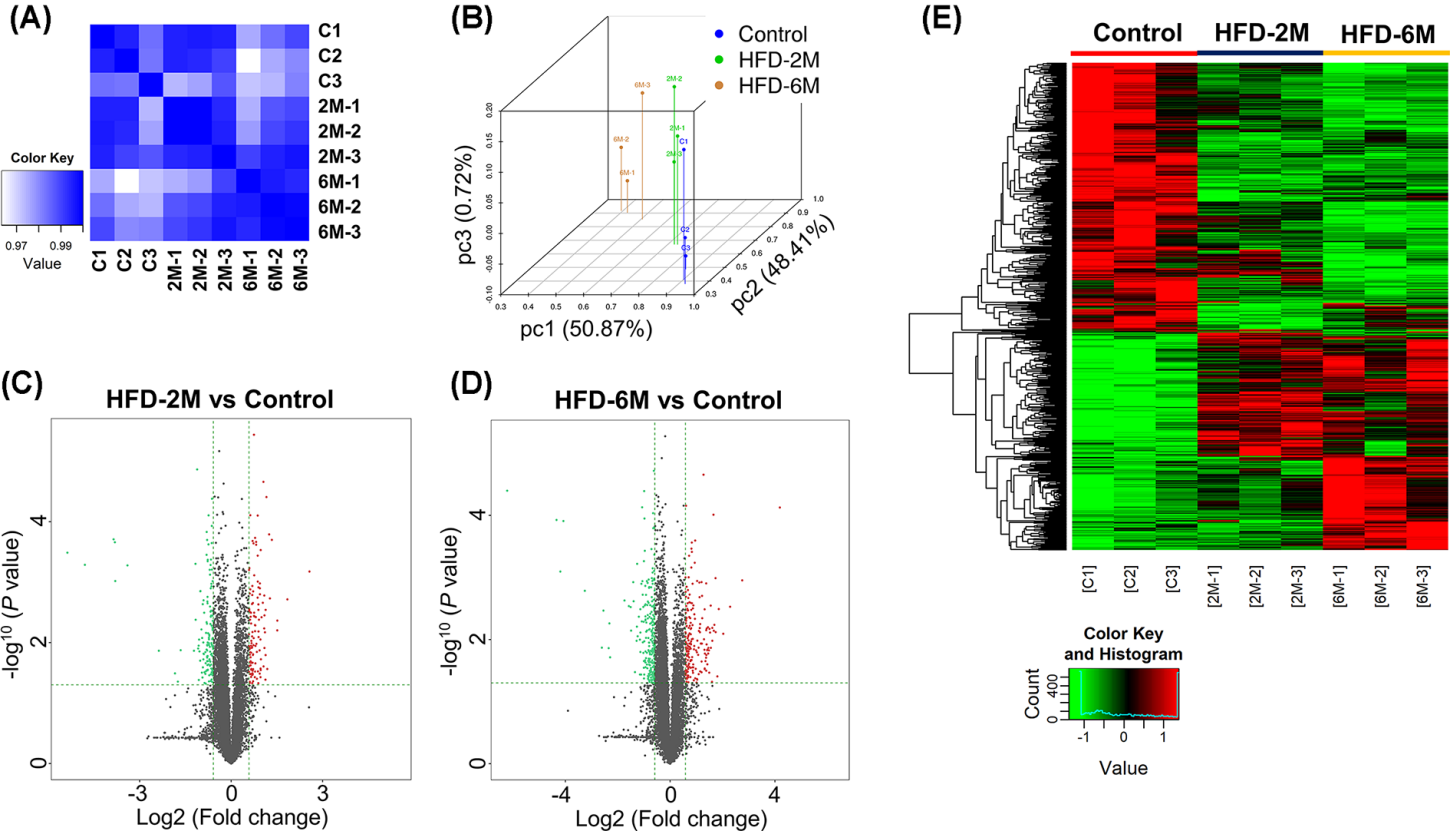
RNA sequencing and analysis (**A**) Pearson correlation coefficient analysis and (**B**) Principal Component Analysis (PCA) of all quantifiable mRNAs from Control, HFD-2M, and HFD-6M islets indicated the reliability of biological replicates. The *X*-axis is pc1, *Y*-axis is pc2, and *Z*-axis is pc3. Differential up-regulated and down-regulated genes in HFD-2M (**C**) and HFD-6M (**D**) groups are shown in Volcano Plot. (**E**) Clustering analysis of gene expression profiles of Control, HFD-2M, and HFD-6M mice are shown in the heat map. PC, principal component.

Next, we collected islets from indicated mice and performed RNA-sequencing. Pearson correlation coefficient analysis showed that the square of Pearson coefficient of biological repeated samples from different groups is more than 0.92, indicating that the reliability between samples is good (Figure 2A). We then performed Principal Component Analysis (PCA) and the result showed that the similarity between samples is good (Figure 2B). As shown in Volcano Plot with screening criteria of log2Fold Change > 1.5 and -log10 *P* value > 0.05, HFD feeding of 2 months resulted in 125 up-regulated genes and 137 down-regulated genes compared with the control group (Figure 2C). In addition, HFD feeding of 6 months resulted in 195 up-regulated genes and 233 down-regulated genes compared with the control group (Figure 2D). A heat map of clustering analysis for three groups was shown in Figure 2E.

**Figure 3:**
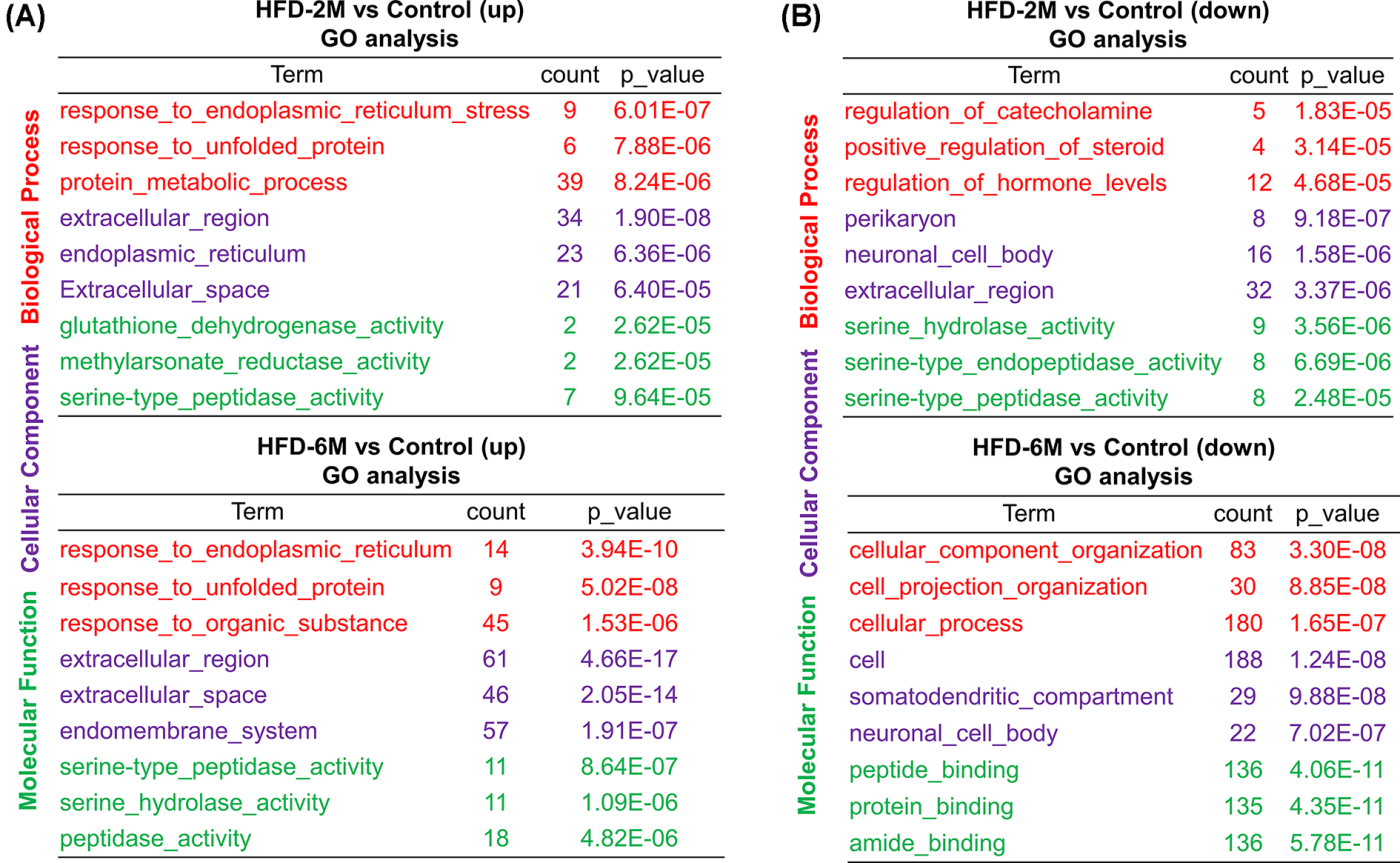
GO enrichment analysis of DEGs (**A**) Up-regulated GO terms in HFD-2M (upper column) and HFD-6M (lower column) group. Significant biological processes are displayed with their respective gene numbers and *P* values. (**B**) Down-regulated GO terms in HFD-2M (upper column) and HFD-6M (lower column) groups. Significant biological processes are displayed with their respective gene numbers and *P* values. Biological process (red); Cellular component (purple); Molecular function (green).

To further study the functional classification of these DEGs, Gene Ontology (GO) analysis was performed. As shown in Figure 3A, biological process-related pathways ‘response to endoplasmic reticulum stress’ and ‘response to unfolded protein’, cellular component-related pathways ‘Extracellular region’ and ‘Extracellular space’ and molecular function-related pathway ‘serine-type peptidase activity’ were up-regulated both in HFD-2M and HFD-6M islets, which is consistent with the previous study [20] reporting that there was excessive endoplasmic reticulum stress in the islets of HFD-induced obese mice. However, in down-regulated DEGs, only the cellular component-related pathway ‘neuronal cell body’ was down-regulated both in HFD-2M and HFD-6M islets (Figure 3B).

**Figure 4:**
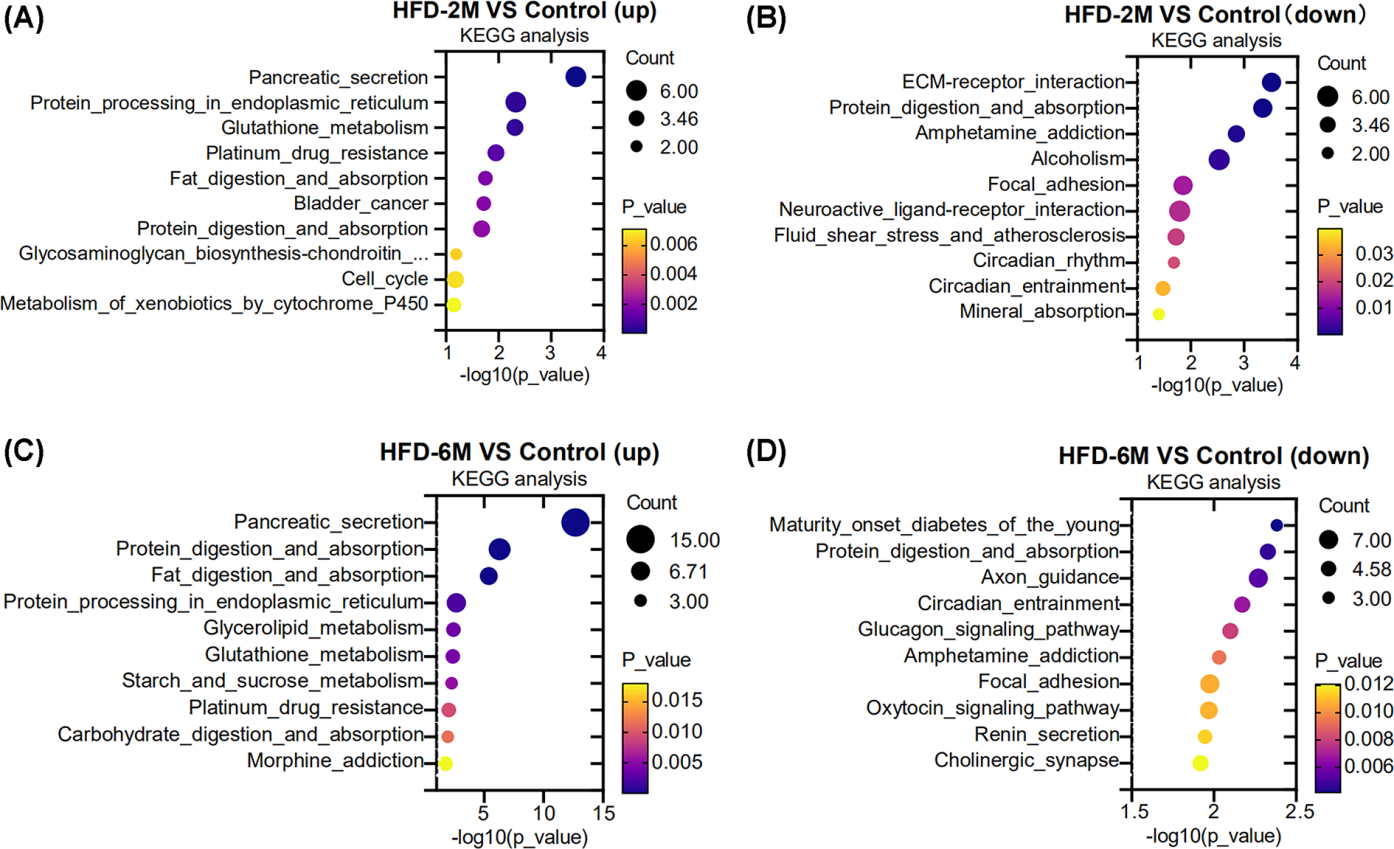
KEGG enrichment analysis of DEGs (**A**) The top 10 up-regulated pathways in the HFD-2M group; (**B**) The top 10 down-regulated pathways in the HFD-2M group. (**C**) The top 10 up-regulated pathways in the HFD-6M group. (**D**) The top 10 down-regulated pathways in the HFD-6M group. The size and color of the bubble represent gene number and *P* value, respectively.

To further study the pathway classification of these DEGs, Kyoto Encyclopedia of Genes and Genomes (KEGG) enrichment analysis was performed and the top 10 differential pathways were shown in Figure 4. Compared with the control group, five pathways including ‘pancreatic secretion’, ‘protein processing in endoplasmic reticulum’, ‘glutathione metabolism’, ‘fat digestion and absorption,’ and ‘protein digestion and absorption’ were up-regulated both in HFD-2M and HFD-6M islets (Figure 4A,C). Whereas in down-regulated pathways, only ‘protein digestion and absorption’, ‘circadian entrainment’ and ‘focal adhesion’ were down-regulated both in HFD-2M and HFD-6M islets (Figure 4B,D).

### Heat map of DEG clusters classified with a differential tendency

**Figure 5:**
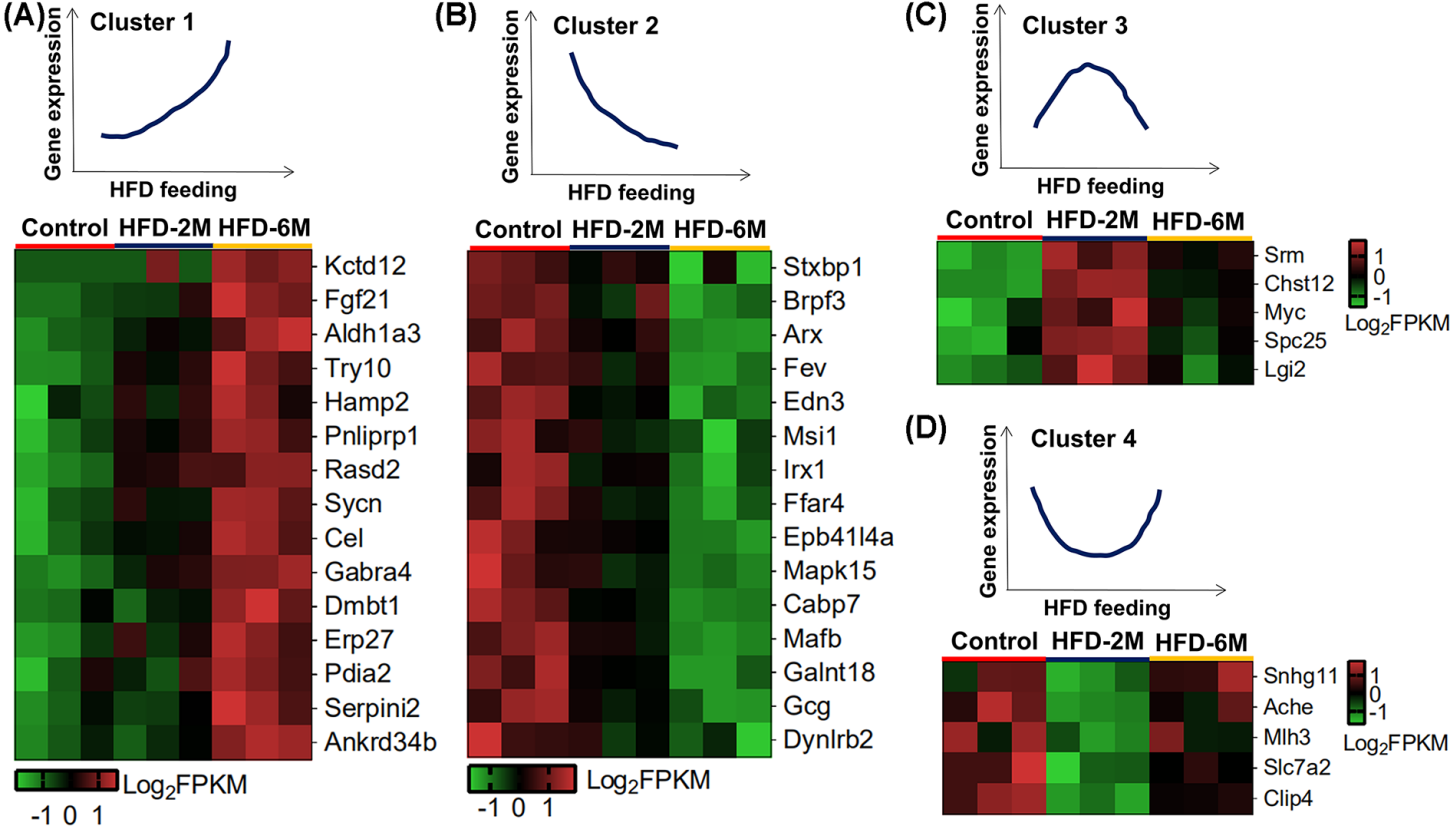
Heat map of DEG clusters classified with differential tendency (**A**) The top 15 genes were up-regulated with the duration of the HFD. (**B**) The top 15 genes were down-regulated with the duration of the HFD. (**C**) The top 5 genes were up-regulated with HFD feeding for 2 months but then down-regulated after 6 months of exposure to HFD. (**D**) The top 5 genes were down-regulated with HFD feeding for 2 months but then up-regulated after 6 months of exposure to HFD.

To investigate the gene expression response to different HFD feeding time, we classified them as different clusters according to their differential tendency. As shown in Figure 5A, the expression of the top 15 DEGs including *Fgf21, Gabra4*, and *Dmbt1* in pancreatic islets were gradually up-regulated along with the HFD feeding. Among them, *Gabra4* has been reported to be associated with T2DM by a discovery of GWAS in United Arab Emirates [21]. In addition, the expression of the top 15 DEGs including α cell markers *Arx, MafB*, and *Gcg* was gradually down-regulated in the pancreatic islets along with the HFD feeding (Figure 5B). Interestingly, *Srm, Chst12, Myc, Spc25*, and *Lgi2* were first up-regulated in the pancreatic islets of mice fed with HFD for 2 months and then down-regulated after 6 months of HFD feeding (Figure 5C). On the contrary, the expressions of *Snhg11, Ache, Mlh3, Slc7a1*, and *Clip4* were firstly down-regulated in islets of mice with 2 months of HFD feeding, then up-regulated after 6 months exposure to HFD (Figure 5D).

### Dedifferentiation of islet cells in HFD-fed mice

**Figure 6:**
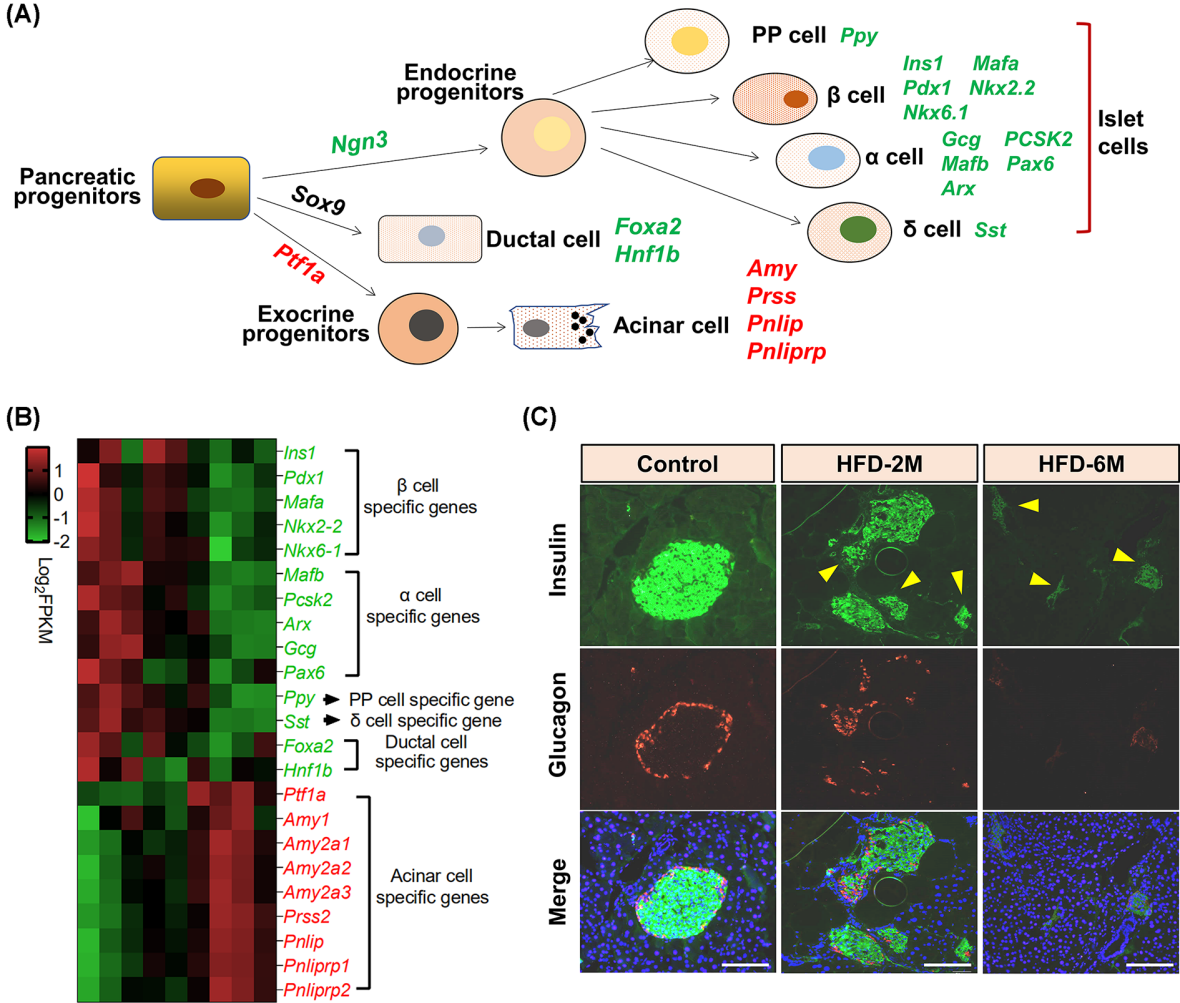
HFD-induced islet cell dedifferentiation in islets (**A**) This illustration shows an overview of the development of pancreatic progenitors, which was modified regarding the figure in the previous research [40]. The master transcription factors within each type of cell are listed; upregulation and downregulation are presented in red and green, respectively. (**B**) Heat map shows master transcription factors within each type of cell in the pancreatic. (**C**) Immunofluorescence staining of insulin and glucagon in the pancreas of Control, HFD-2M, and HFD-6M mice. green: insulin, red: glucagon, blue: DAPI. The bar represents 100μm.

Given that there are exocrine cells like acinar cells, ductal cells, and islet cells in the pancreas, and the islet cells are composed of α cells, β cells, δ cells, and pancreatic polypeptide cells (PP cells) (Figure 6A) [22], we next assessed the effect of HFD feeding on the marker gene expression of these different cell types in the pancreas. As shown in Figure 6B, *Ngn3*, a marker of pancreatic endocrine progenitor cells, was significantly down-regulated in the pancreatic islets of HFD-fed mice. Similarly, genes functionally associated with β cells, including *Ins1, Pdx1, Nkx2.2, Nkx6.1, and Mafa* were also down-regulated as well as genes specifically expressed in α cells, including *Gcg, PCSK2, Mafb, Pax6*, and *Arx*). Notably, δ cells specifically expressed *Sst* and PP cells specifically expressed *Ppy* were also remarkably down-regulated in islets of HFD-fed mice. These data indicate that HFD feeding led to the dedifferentiation of islet cells. Interestingly, *Ptf1a*, a marker of pancreatic exocrine progenitor cells, was significantly up-regulated in the HFD-fed islets as well as the acinar cell specifically expressed genes *Amy, Press, Pnlip, and Pnliprp*. Besides, two genes associated with the differentiation and development of ductal cells *Foxa2* and *Hnf1b* were also down-regulated. To further confirm the mRNA levels of islet cell-related genes in RNA-seq, we next performed immunostaining to detect the protein levels of Glucagon and Insulin in the pancreas from control or HFD-fed mice (Figure 6C). As expected, we found that the protein levels of Insulin and Glucagon were reduced along with HFD feeding. Taken together, these data suggest that HFD feeding induces the dedifferentiation of islet cells with decreased endocrine cell markers and increased exocrine cell markers.

### HFD feeding induced reduction of collagen gene expression in islets

**Figure 7:**
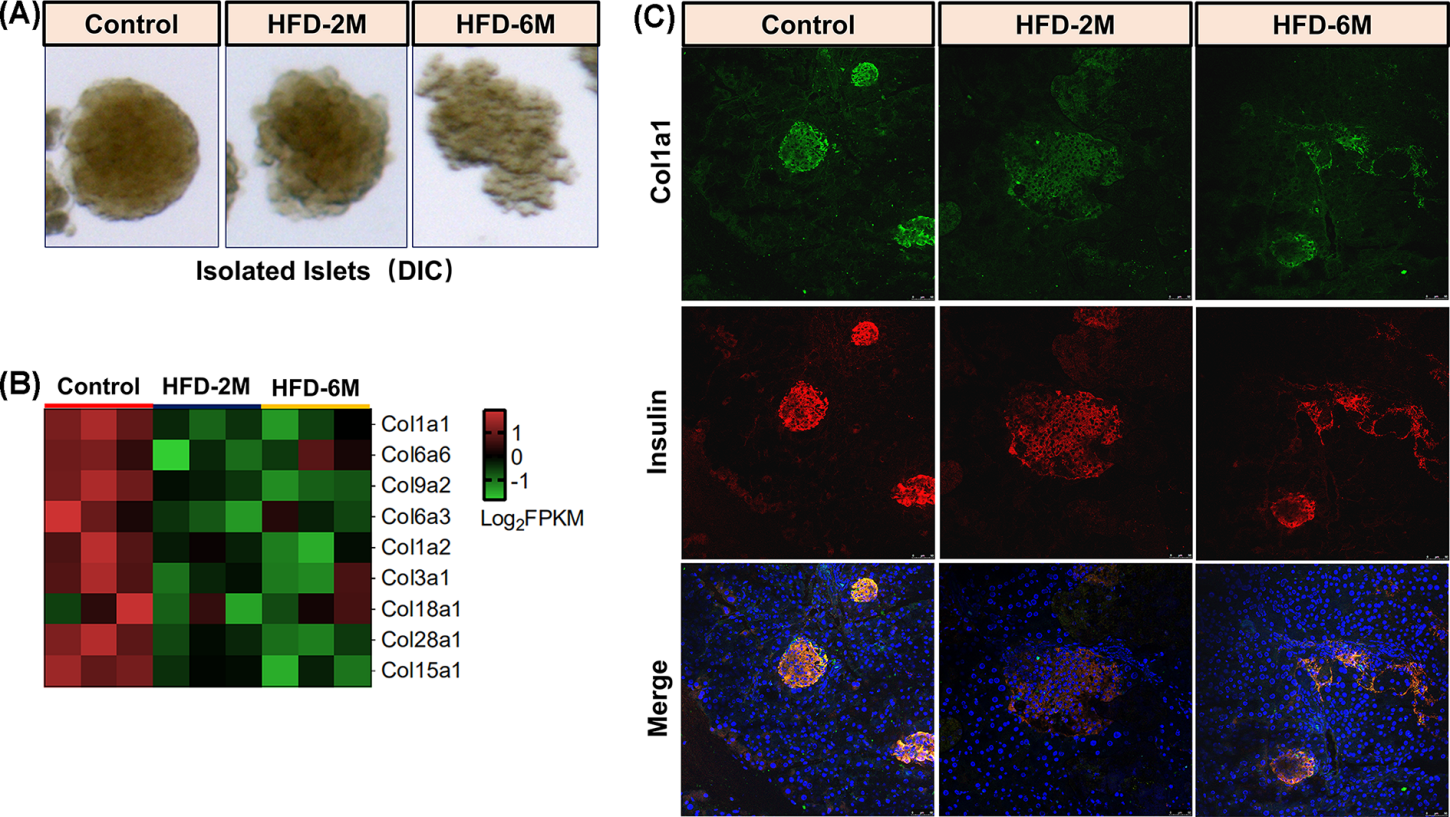
HFD induced down-regulation of collagen family in islets (**A**) DIC representative islets images of Control, HFD-2M, and HFD-6M mice. (**B**) The heat map shows collagen family was downregulated in mice islets with the HFD feeding. (**C**) Immunofluorescence staining of *Col1a1* and insulin in the pancreas of Control, HFD-2M, and HFD-6M mice. green: *Col1a1*, red: insulin, blue: DAPI. The bar represents 100μm.

Compared with the control group, the islets of mice induced by HFD for 2 months showed a slight morphological defect, while 6 months of HFD feeding led to obvious irregular deformation of the islets (Figure 7A). Given that the collagen family, the main component of extracellular matrix (ECM), are important in the morphological changes of islets [23], we further analyzed the expression of genes belong to collagen family. Notably, 9 genes of the collagen family, including *Col1a1, Col6a6, Col9a2, Col6a3, Col1a2, Col3a1, Col18a1, Col28a1*, and *Col15a1*, were down-regulated in the HFD-2M and HFD-6M group (Figure 7B). Furthermore, we performed immunofluorescence staining to detected the protein level of one of the collagen family *Col1a1* and found that fluorescence signal of Col1a1 *protein* was getting weaker along with HFD feeding.

## Discussion

For a long time, obesity and T2DM have seriously affected human health and greatly increased the social and economic burden. Insulin resistance and islet β-cell dysfunction are the core factors of the progression of T2DM [24,25]. However, the molecular mechanism of the structural and functional changes of pancreatic islets during the progression from obesity to T2DM remains to be determined. Therefore, the key point of current research is to explore the impact of obesity on pancreatic islets and provide new prevention and treatment strategies for the damage of pancreatic islets and the development of T2DM.

In this study, we established a mouse model of HFD-induced obesity and β cell dysfunction for a transcriptome sequencing analysis of the pancreatic islets at different stages of obesity. We found that HFD feeding severely damaged the morphological structure of pancreatic islets and the number and secretory function of β cells, which is very similar to the development of β-cell dysfunction in human T2DM [26]. The large-scale transcriptome analysis revealed sufficient coverage depth and quantitative accuracy to generate functional maps of healthy and diseased islets in unprecedented detail. Many previously identified DEGs and pathways were confirmed in this study, such as *Fgf21* [27], *Ffar4* [28], *Myc* [29], pancreatic secretion, fat digestion and absorption [13], and endoplasmic reticulum stress [20], which verified the analytical method of this study. More importantly, the construction of gene expression profiles based on the time course enabled us to determine the process of biological events leading to obese islet injury.

Interestingly, we found the differential tendency of some DEGs is not as regular as that in Figure 5A,B, indicating that some DEGs exhibited different transcriptional response to HFD-2M and HFD-6M. To explain this, we supposed that there are some protective mechanisms which are triggered by short-term HFD feeding (HFD-2M) with increase of protective genes and decrease of negative genes, but impaired by long-term HFD feeding (HFD-6M) with decrease of protective genes and increase of negative genes.

For example, in Figure 5C, *Myc* have been reported to be elevated and required for increased pancreatic β-cell replication and expansion during metabolic stress induced by short-term high-fat diet (HFD) in young mice [30], which is consistent with our observation. Then the decrease of *Myc* in HFD-6M might due to the impairment of the protective mechanism which triggered the *Myc* expression. As to another gene, *Srm*, the inhibition of which was reduces β cell mass [31], it is adaptively increased after short-term HFD feeding as a protective gene and decreased after long-term HFD feeding with the destruction of the adaptively protective mechanism. In a word, in Figure 5C, protective genes were adaptively increased after short-term HFD feeding and decreased after long-term HFD feeding with the destruction of the adaptively protective mechanism. In Figure 5D, *SNHG11*, which was up-regulated in plasma of chronic pancreatitis (CP) patients and TGF-β1-treated pancreatic stellate cells (PSCs) [32], was adaptively decreased after short-term HFD feeding as a negative gene and increased after long-term HFD feeding with the destruction of the adaptively protective mechanism. *Ache* (acetylcholinesterase) is responsible for hydrolysis of acetylcholine which is reported to stimulate the insulin secretion of β cells [33]. Moreover, *Ache* expression is up-regulated in STZ-induced diabetic mouse model and promotes apoptosis of β cells [34]. As a negative gene, Ache was adaptively decreased after short-term HFD feeding as a negative gene and increased after long-term HFD feeding with the destruction of the adaptively protective mechanism.

Our study suggests that the reduction in β-cell mass and the shift in islet morphology likely contribute to islet injury in HFD-induced obese mice. Many islet-function-related transcriptional factors, e.g., *Pdx1, Pax6*, and *Mafa*, required for the specification of endocrine cells were downregulated in HFD-induced obese mice, whereas those required for exocrine cells were upregulated (Figure 6). Among these transcriptional factors, the overexpression of *Pdx1, Pax6*, and *Mafa* has also been reported to preserve β-cell function under T2DM conditions [35–37]. We found that many genes required for β cell proliferation were significantly down-regulated, suggesting that the proliferation of β cells is defective in the process of obesity induced by HFD. Another significant feature of our data is the observation of a change in islets morphology,

Collagens are deposited in the extracellular matrix, and the changes in the pancreatic extracellular matrix are related to the pathogenesis of diabetes, including the loss of the basement membrane and integrity of the interstitial matrix. Wang et al. [38] reported that the collagen capsule was fragmented around the islets of T1DM compared with the control islets surrounded by collagen. *Col5a3*, a member of the collagen family, is crucial to the glucose homeostasis of mice. Researchers have demonstrated that *Col5a3* knockout in mice leads to the reduction of islet numbers and β cell function decreased [39]. Interestingly, our results found that nine coding genes (*Col1a1, Col6a6, Col9a2, Col6a3, Col1a2, Col3a1, Col18a1, Col28a1*, and *Col15a1*) of the collagen family were significantly downregulated in the HFD-induced obese mice islets (Figure 7), suggesting that collagen plays an important role in maintaining the normal morphology and function of the islets, and the β cell microenvironment may be a key driver leading to the eventual deterioration of islet function and β cell decompensation.

## Conclusions

We have established an HFD-induced obese mouse model, summarized the dynamic changes in the morphology and function of pancreatic islet tissue during the process of obesity to T2DM, and demonstrated the loss of collagen in pancreatic islets during this process. Collectively, our study suggests that the collagen family may be a key target for the prevention and treatment of islet injury in obesity and T2DM, which provides important clues for the clinical treatment of islet injury in obesity.

## Data Availability

The datasets used and analyzed in the present study are available from the corresponding author.

## Abbreviations

CP: Chronic pancreatitis
DEG: Differentially expressed gene
HFD: High-fat diet
MetS: Metabolic syndrome
OGTT: Oral glucose tolerance test
PCA: Principal Component Analysis
PSC: Pancreatic stellate cell
T2DM: Type 2 diabetes mellitus
